# Differences between young and older adults in physiological and subjective responses to emotion induction using films

**DOI:** 10.1038/s41598-020-71430-y

**Published:** 2020-09-03

**Authors:** Luz Fernández-Aguilar, José M. Latorre, Arturo Martínez-Rodrigo, José V. Moncho-Bogani, Laura Ros, Pablo Latorre, Jorge J. Ricarte, Antonio Fernández-Caballero

**Affiliations:** 1 Department of Psychology, University of Castilla La Mancha, Albacete, Spain; 2 Department of Informatic Systems, University of Castilla La Mancha, Albacete, Spain; 3 Department of Medical Sciences, University of Castilla La Mancha, Albacete, Spain; 4 Institute for Research in Biomedicine (IRB Barcelona), The Barcelona Institute of Science and Technology, Barcelona, Spain; 5 Departament of Ciències Experimentals I de La Salut, Cell Signaling Research Group, Universitat Pompeu Fabra (UPF), Barcelona, Spain; 6 Neurological Disabilities Research Institute (IDINE), Albacete, Spain

**Keywords:** Psychology, Health care

## Abstract

Emotional response in aging is typically studied using the dimensional or the discrete models of emotion. Moreover, it is typically studied using subjective or physiological variables but not using both perspectives simultaneously. Additionally, tenderness is neglected in emotion induction procedures with older adults, with the present work being the first to include the study of physiological tenderness using film clips. This study integrated two separate approaches to emotion research, comparing 68 younger and 39 older adults and using a popular set of film clips to induce tenderness, amusement, anger, fear, sadness and disgust emotions. The direction of subjective emotional patterns was evaluated with self-reports and that of physiological emotional patterns was evaluated with a wearable emotion detection system. The findings suggest a dual-process framework between subjective and physiological responses, manifested differently in young and older adults. In terms of arousal, the older adults exhibited higher levels of subjective arousal in negative emotions and tenderness while young adults showed higher levels of physiological arousal in these emotions. These findings yield information on the multidirectionality of positive and negative emotions, corroborating that emotional changes in the adult lifespan appear to be subject to the relevance of the emotion elicitor to each age group.

It is widely recognized that aging is associated with changes in the direction of emotional patterns^1^, but the knowledge of how the processes involved in emotions change as we age is still incomplete^2^. In recent decades, much work has been done to establish different theories to explain emotional aging. Emotional theories such as the Socioemotional Selectivity Theory (SST^3^; have underlined a positivity effect, whereby older adults tend to give primacy to achieving emotional gratification^1^, and avoid or mitigate exposure to negative situations^4,5^. This model has been empirically studied in psychological and neuroscientific research^2,6,7^ and is currently one of the most widely accepted models. However, Schweizer et al.^8^ found that older adults exhibited decline in the capacity to down-regulate negative affect with film clips, which would suggest an absence of the positivity effect. Other authors have suggested that differences in the emotional experience depend on the stimuli used in the Mood Induction Procedure (MIP)^9^. For example, different studies have found that older adults present greater emotional responses when the eliciting stimuli involve irrevocable personal loss or deal with social injustice^10,11^.

Compared with the literature on subjective emotional patterns, that on physiological patterns in aging is limited. A meta-analytical review of 31 studies using different MIPs conducted in laboratory settings found that the young and older adults’ physiological patterns were similar, although the amplitude of this response was attenuated in old age^12^. In addition, studies using film clips to elicit emotions have reported reduced autonomic response in older participants compared to younger participants^13^ and higher maximum heart rates in young adults^14^. In contrast, Seider et al.^10^ found that, on both self-report and physiological measures, older participants showed higher levels in response to clips eliciting sadness. In this sense, some authors have suggested that emotion regulation might be more difficult in response to higher-intensity stimuli^15^, while others have found no differences in emotional experience between young and older adults^16^.

To understand emotional processing, it is important to consider the role of multiple responses. Emotion research has primarily focused on examining emotional valence and arousal, while few studies have conducted an analysis jointly using the dimensional and discrete models of emotion^17^. Although the classification of discrete emotions may be facilitated by the differences in valence and arousal^18,19^, no previous study has focused on discrete and dimensional emotional models simultaneously in old age. This study focuses on both emotional models considering the subjective experience and autonomic patterns of emotions. It is necessary to study autonomic responses to understand the emotions. For example, electrodermal activity (EDA) reports on the level of arousal of emotional stimuli^17^ and heart rate (HR) variability is a potential indicator of emotional valence^20^. Although the literature recommends using different means to determine the effectiveness of the MIP in inducing the expected emotions^21–24^, empirical evidence for the association and coherence between the subjective and physiological emotional response in older adults is scarce and inconsistent^25^.

With regard to the stimuli selected to induce emotions, film clips is one of the most widely used and effective laboratory MIPs (see Rottenberg et al.^22^ for a review). However, few studies have focused on examining the effectiveness of this procedure in older adults yielding different results. For example, Beaudreau et al.^14^ were the first to examine emotional response of older adults with a standardized set of film clips depicting amusement, fear, anger and sadness. They found similar responses in younger and older adults except for anger, where older adults exhibited higher levels on the target emotion and non-target negative emotions. A later study also reported similar results but older adults showed higher global intensity for the clips inducing fear, anger, disgust, and sadness^26^. Regarding positive emotions, other researchers found that older adults showed higher levels of positive emotions^27^. This finding was supported in a recent study including the target of tenderness. However, young participants showed a significantly higher level of pleasantness in response to amusement clips^28^.

Currently, several questions on the study of emotions in aging remain unanswered. Therefore, this study focuses on exploring the interactions of subjective and autonomic responses of older adults’ emotions, considering dimensional and discrete measures^29,30^ and using a contemporary film stimuli set (see Fernández-Aguilar et al.^28^ for a review of set). This set have shown to be an effective tool in young adults^31^. Nevertheless, the present study is the first to examine the directions of physiological and subjective emotional patterns of older adults with this popular set of films. In addition, this set includes the target emotion of tenderness. Despite tenderness not being considered a basic emotion, the interest here is that attachment-related emotions are of great importance in biological, emotional and social human development. This emotion is not usually included in MIPs and, to our knowledge, past research has not attempted to study the physiological response with tenderness as a target in an MIP with older adults. The literature on emotional psychophysiology has traditionally focused on studying the autonomic response to negative emotions, largely neglecting the study of positive emotions. Shiota et al.^32^, however, analyzed the autonomic response associated with positive emotions, finding changes occurred in the autonomic nervous system depending on the emotional target. For example, when eliciting nurturant love, an increase in heart rate was observed, while amusement failed to generate the expected cardiovascular arousal. The present study aims to extend scientific knowledge on the peculiarities of the autonomic response to various positive emotions.

The main goal of this research is to analyze the interaction between psychological and physiological components of positive and negative emotions in older adults, compared to their younger counterparts. To do this, we use self-reports and SCL and HR considering dimensional and discrete models of emotion. Thus, we register the autonomic response patterns while watching the clips and the subjective emotional patterns after each clip to determine the association between the two emotional system processes.

Drawing on the above, we hypothesize that film clips are an effective MIP for eliciting positive and negative emotions in both younger and older adults. We expect to find no differences between younger and older participants in physiological arousal (EDA). However, we predict differences in the intensity of the emotional response, in particular, with older adults exhibiting lower intensity in their responses^12^. Regarding subjective measures, we expect to find differences between the two groups. Finally, we expect the older adults to report lower intensity and lower negative mood at both the evaluation and interpretation stages in response to the unpleasant stimuli.

## Results

### Assessment of the baseline state

The analyses based on the baseline state of the participants showed no differences between age groups, except for arousal. Specifically, the younger participants showed greater subjective arousal [*t*(105) = 3.45, *p* = .001, *d* = 0.69] and greater physiological arousal (SCL) [*t*(105) = 2.67, *p* = .009, *d* = 0.51] compared with the older adults. These results suggest that, before starting the mood induction procedure, the older adults presented lower emotional arousal than the younger participants.

### Ability to identify the target emotions

**Figure 1:**
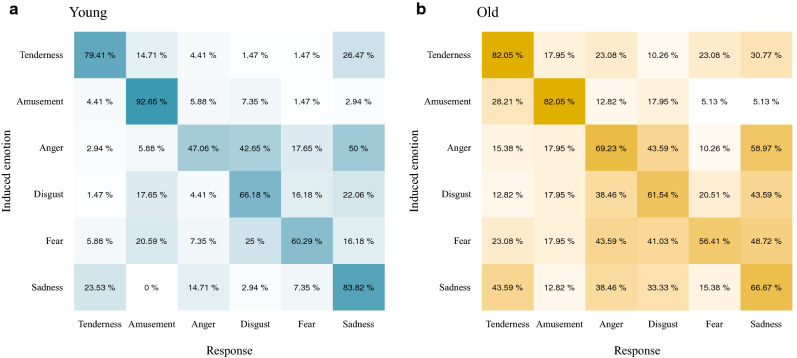
Young and older adults’ ratings of emotional targets and non-target response to the target films. ggplot graphs.

The discriminant analysis (DA) based on the ability to identify the target emotions showed that the young adults correctly classified a higher percentage of all negative emotions [disgust = 94.12, fear = 89.71, anger = 89.71, sadness = 83.82, total = 95.59] than the older adults [disgust = 43.59, fear = 51.28, anger = 33.33, sadness = 66.67, total = 87.18]. Likewise, the young adults correctly classified a higher percentage of all positive emotions [amusement = 91.18, tenderness = 95.59, total = 89.71] than the older adults [amusement = 38.46, tenderness = 28.21, total = 56.41]. The results show that the older adults’ responses were more varied as regards the number of emotions they identified for a particular target (see Fig. 1). Although both groups correctly identified each emotion, our results reveal differences in the level of identification. For the positive emotions, amusement was experienced more intensely by the young adults than the older adults [*t*(105) = 2.46, *p* = .015, *d* = 0.49]. In the case of negative emotions, fear was experienced more intensely by the younger adults than the older adults [*t*(105) = 2.87, *p* = .005, *d* = 0.58].

### Effect of the emotion induction and age

To analyze the effects of the emotion induction, age group, and age × emotion interaction, Table 1 shows the results separately organized for each of the six target emotions.

**Table 1:** Subjective and physiological emotion patterns differences in emotional targets between young and old adults.

Emotion/measure	Young adults (*n* = 68)	Old adults (*n* = 39)	*F* age		*F* emotion		*F* age × emotion	
	*M* (*SD*)	*M* (*SD*)	*F*(1,105)	*η*^2^	*F*(1,105)	*η*^2^	*F*(1,105)	*η*^2^
Tenderness								
Valence SAM	6.43 (2.51)	6.28 (2.80)	0.58	.00	21.36**	.16	1.76	.01
Arousal SAM	4.91 (2.18)	5.00 (2.77)	3.17	.02	34.41**	.24	6.33*	.05
Tenderness DES	5.54 (1.84)	5.90 (1.60)	2.97	.02	310.17**	.74	0.03	.00
SCL	597.59 (222.04)	482.51 (172.96)	8.49**	.07	46.93**	.31	1.52	.01
HR	81.64 (10.40)	79.40 (10.02)	0.27	.00	59.51**	.37	4.05*	.03
Amusement								
Valence SAM	5.88 (2.26)	5.69 (2.83)	0.43	.00	11.47**	.09	2.87	.02
Arousal SAM	4.49 (2.20)	3.26 (2.26)	15.71**	.13	3.80*	.03	0.01	.00
Amusement DES	5.19 (1.53)	4.38 (1.77)	5.43	.04	258.92**	.71	2.97	.02
SCL	590.05 (207.70)	503.69 (193.27)	6.16*	.05	52.51**	.34	0.09	.00
HR	82.37 (11.12)	77.72 (8.19)	1.10	.01	46.68**	.31	22.23**	.18
Anger								
Valence SAM	2.07 (1.82)	2.15 (2.15)	1.83	.01	99.30**	.48	1.14	.01
Arousal SAM	6.04 (2.19)	6.41 (2.39)	2.14	.02	109.90**	.51	9.07**	.08
Anger DES	4.85 (1.87)	5.21 (2.15)	1.21	.01	304.54**	.74	0.20	.00
SCL	595.27 (224.90)	468.29 (128.66)	10.90**	.10	33.63**	.25	1.58	.01
HR	82.20 (11.02)	79.04 (9.82)	0.69	.00	39.18**	.28	5.33**	.05
Disgust								
Valence SAM	2.50 (1.77)	1.82 (1.62)	0.00	.00	98.46**	.48	6.02**	.05
Arousal SAM	5.76 (2.08)	5.79 (2.69)	3.59	.03	84.84**	.44	6.23**	.05
Disgust DES	4.84 (1.91)	4.62 (2.35)	0.33	.00	288.68**	.73	0.20	.00
SCL	578.51 (183.57)	488.75 (188.23)	6.95**	.06	43.04**	.30	0.43	.00
HR	82.18 (11.09)	79.06 (9.06)	0.62	.00	60.77**	.37	6.79**	.06
Fear								
SAM valence	3.04 (1.95)	2.54 (1.89)	0.10	.00	56.37**	.34	4.41*	.04
SAM arousal	6.93 (1.88)	5.51 (2.66)	16.30**	.13	148.67**	.58	0.07	.00
CED fear	4.49 (2.04)	3.28 (2.15)	4.57*	.04	138.17**	.56	9.86**	.08
SCL	609.15 (205.92)	489.57 (163.31)	9.62**	.08	59.34**	.37	2.64	.02
HR	84.24 (10.79)	78.99 (10.14)	2.07	.02	76.21**	.43	15.18**	.13
Sadness								
Valence SAM	2.87 (1.64)	2.64 (2.19)	0.71	.00	59.45**	.36	2.63	.02
Arousal SAM	5.00 (1.92)	5.38 (2.48)	1.90	.01	60.04**	.36	12.40**	.10
Sadness DES	5.41 (1.51)	5.13 (1.90)	0.38	.00	243.63**	.69	0.39	.00
SCL	569.80 (184.22)	535.74 (193.89)	5.98*	.05	33.23**	.24	1.01	.01
HR	82.34 (11.19)	79.02 (8.76)	0.69	.00	85.74**	.45	10.66**	.09

* *p* ≤ .050; **p ≤ .010.

With respect to the effectiveness of the MIP (emotion factor), exposure to the six emotions generated significant changes in all the subjective and physiological variables, regardless of age group. Effect sizes range from a minimum of *η*^2^ = .03 to a maximum of *η*^2^ = .74, with most values being above *η*^2^ = .30 (large effect size). The greatest effects were found for the variables related to emotion identification evaluated on the DES.

### Effect of negative emotion induction

The two-way ANOVA (neutral induction vs. anger induction × young adults vs. older adults; neutral induction vs. disgust induction × young adults vs. older adults; neutral induction vs. fear induction × young adults vs. older adults; neutral induction vs. sadness induction × young adults vs. older adults) shows a main effect of age group on the SCL variable: the young adults presented higher levels of skin conductance compared to the older adults. In the direction of HR pattern, we also found significant effects for the age group × emotion interaction, where the young adults (anger: [*t*(67) =  − 6.17, *p* ≤ .001, Cohen’s *d* = 0.47]; disgust: adults [*t*(67) =  − 7.98, *p* ≤ .001, Cohen’s *d* = 0.50]; fear: [*t*(67) =  − 10.08, *p* ≤ .001, Cohen’s *d* = 0.66]; sadness: [*t*(67) =  − 9.69, *p* ≤ .001, Cohen’s *d* = 0.51]) showed a greater increase in their response than the older adults (anger: [*t*(38) =  − 3.68, *p* ≤ .001, Cohen’s *d* = 0.35]; disgust: [*t*(38) =  − 3.79, *p* ≤ .001, Cohen’s *d* = 0.30]; fear: [*t*(38) =  − 3.19, *p* = .003, Cohen’s *d* = 0.28]; sadness: [*t*(38) =  − 4.29, *p* ≤ .001, Cohen’s *d* = 0.30]).

**Figure 2:**
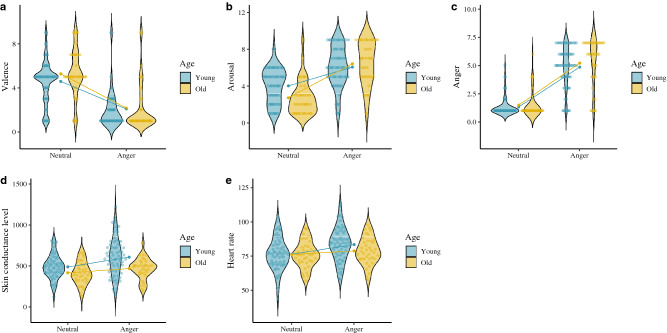
Subjective and physiological response by target condition and age group. (**A**) Anger valence (SAM). (**B**) Anger arousal (SAM). (**C**) Anger emotion level (CED). (**D**) Anger skin conductance level. (**E**) Anger heart rate. ggplot graphs.

**Figure 3:**
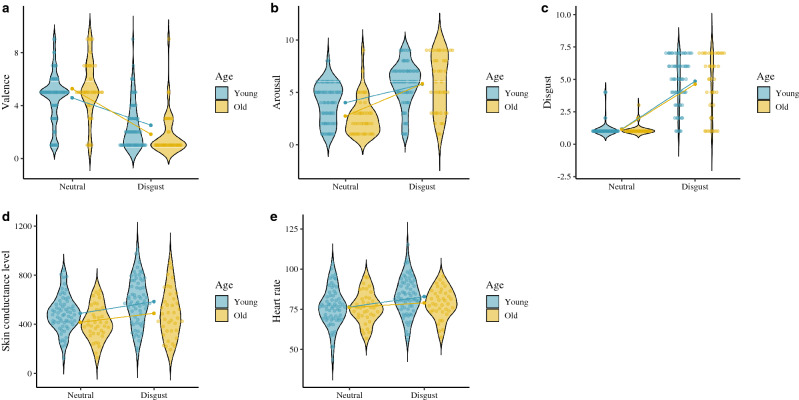
Subjective and physiological response by target condition and age group. (**A**) Disgust valence (SAM). (**B**) Disgust arousal (SAM). (**C**) Disgust emotion level (CED). (**D**) Disgust skin conductance level. (**E**) Disgust heart rate. ggplot graphs.

**Figure 4:**
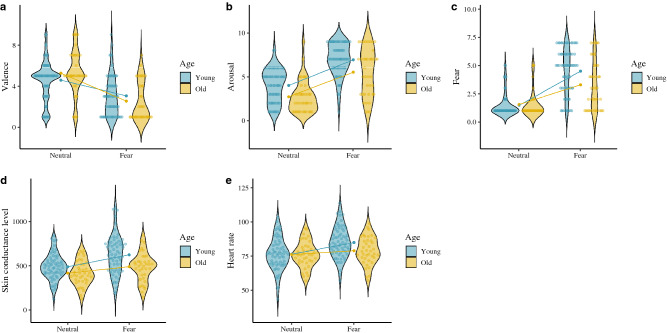
Subjective and physiological response by target condition and age group. (**A**) Fear valence (SAM). (**B**) Fear arousal (SAM). (**C**) Fear emotion level (CED). (**D**) Fear skin conductance level. (**E**) Fear heart rate. ggplot graphs.

**Figure 5:**
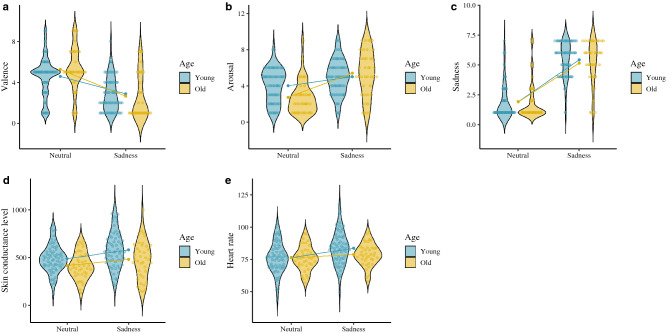
Subjective and physiological response by target condition and age group. Note: (**A**) Sadness valence (SAM). (**B**) Sadness arousal (SAM). (**C**) Sadness emotion level (CED). (**D**) Sadness skin conductance level. (**E**) Sadness heart rate. ggplot graphs.

Respect to the subjective variables, we found significant effects for the age group × emotion interaction on anger, disgust and sadness targets, where the older adults (anger: [*t*(38) =  − 7.99, *p* ≤ .001, Cohen’s *d* = 1.73]; disgust: [*t*(38) =  − 6.21, *p* ≤ .001, Cohen’s *d* = 1.33]; sadness: [*t*(38) =  − 5.92, *p* ≤ .001, Cohen’s *d* = 1.22]) reported a greater increase in their subjective arousal than the young adults (anger: [*t*(67) =  − 6.41, *p* ≤ .001, Cohen’s *d* = 1.00]; disgust: [*t*(67) =  − 6.31, *p* ≤ .001, Cohen’s *d* = 0.89]; sadness: [*t*(67) =  − 4.01, *p* ≤ .001, Cohen’s *d* = 0.53]). However, we found a main effect of age on the subjective evaluation of fear arousal, which the young adults scored higher on subjective arousal than he older adults. With regard to valence, we found a significant effect of the age × emotion interaction on disgust and fear targets, where the older adults (disgust: [*t*(38) = 7.11, *p* ≤ .001, Cohen’s *d* = 1.80]; fear: [*t*(38) = 5.76, *p* ≤ .001, Cohen’s *d* = 1.34]) scoring lower on subjective valence compared to their younger counterparts (disgust: [*t*(67) = 6.55, *p* ≤ .001, Cohen’s *d* = 1.12] ; fear: [*t*(67) = 4.60, *p* ≤ .001, Cohen’s *d* = 0.79]). Finally, we found the age × emotion interaction on target emotion identification (DES), with older adults scoring lower on the identification of this emotion [*t*(38) =  − 4.92, *p* ≤ .001, Cohen’s *d* = 1.00] compared to the young adults [*t*(67) =  − 13.10, *p* ≤ .001, Cohen’s *d* = 1.87]. These findings suggest that despite physiological patterns (SCL and HR) being higher in the young adults, the older adults interpreted that their arousal was more intense. However, in the case of fear target, the older participants rated it higher on unpleasantness and found the target emotion more difficult to identify than the young adults. Accordingly, fear appears to have a greater subjective and physiological impact on the young adults. See Figs. 2, 3, 4 and 5.

### Effect of positive emotion induction

**Figure 6:**
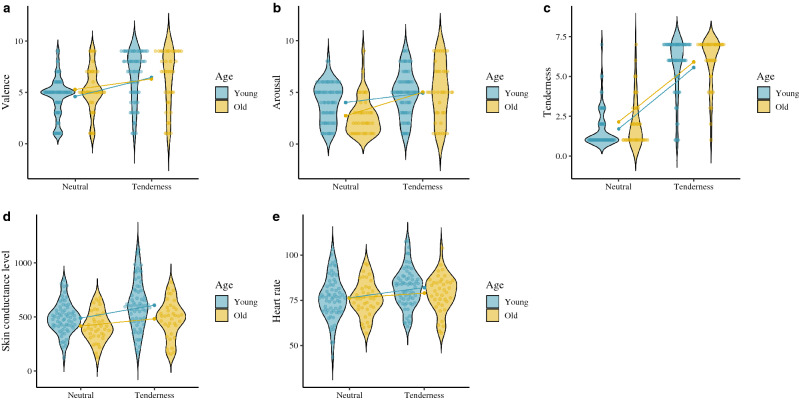
Subjective and physiological response by target condition and age group. (**A**) Tenderness valence (SAM). (**B**) Tenderness arousal (SAM). (**C**) Tenderness emotion level (CED). (**D**) Tenderness skin conductance level. (**E**) Tenderness heart rate. ggplot graphs.

**Figure 7:**
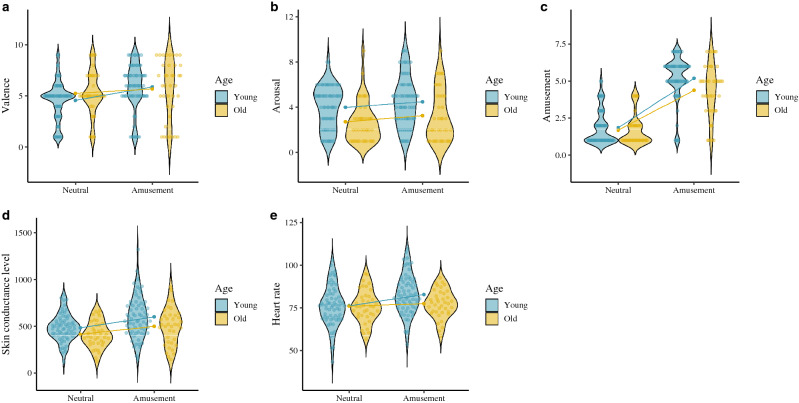
Subjective and physiological response by target condition and age group. (**A**) Amusement valence (SAM). (**B**) Amusement arousal (SAM). (**C**) Amusement emotion level (CED). (**D**) Amusement skin conductance level. (**E**) Amusement heart rate. ggplot graphs.

The two-way ANOVA (neutral induction vs. tenderness induction × young adults vs. older adults; neutral induction vs. amusement induction × young adults vs. older adults) showed a main effect of age on SCL, with the young adults exhibiting higher skin conductance than the older participants. We also found a significant effect of the emotion × age interaction on HR. In particular, the young group (*t*(67) =  − 7.70, *p* ≤ .001, Cohen’s *d* = 0.49) exhibited higher HR than the older group (*t*(38) =  − 3.83, *p* ≤ .001, Cohen’s *d* = 0.32) on tenderness target. In the case of amusement, the young adults’ HR increasing (*t*(67) =  − 9.18, *p* ≤ .001, Cohen’s *d* = 0.51), while the older adults showed no change in this variable (*t*(38) =  − 1.44, *p* = .156, Cohen’s *d* = 0.12).We also found significant effects of the age × emotion interaction on the subjective evaluation of arousal. The older adults [*t*(38) =  − 5.04, *p* ≤ .001, Cohen’s *d* = 0.92] scored higher on the subjective arousal scale compared to the young adults [*t*(67) =  − 2.84, *p* = .006, Cohen’s *d* = 0.45] on tenderness emotion. Respect to the amusement, we found a main effect of age on the variables of subjective arousal and target emotion identification (DES), indicating that the younger adults experienced higher levels of subjective arousal and identified the target emotions with greater intensity compared to their older counterparts. These findings suggest that, on the one hand, amusement emotion has a greater impact on young adults compared to older ones, at both a subjective and physiological level. On the other hand, the subjective arousal of tenderness has a greater impact on the older adults, although the autonomic response is greater in young adults. See Figs. 6 and 7.

## Discussion

Adopting a popular set of film clips^28,31^, the present study is the first to explore both subjective and autonomic emotional response in older people with this tool. Film clips generated significant changes at both subjective and physiological emotional levels in both age groups. For neutral clips, no significant differences being found in the subjective and physiological measures between young and older adults, except for the arousal. Consequently, the results suggest that the differences in negative and positive emotional responses were due to the MIP and not to the previous emotional state^28,33^.

Our findings confirmed that young and older adults present different patterns of response to negative stimuli. However, within each age group, similar responses were found across the different emotions. For example, the responses to the disgust and anger stimuli followed a similar pattern: young adults exhibited a more intense physiological level (SCL and HR) but the older adults reported greater arousal. Although both subjective and physiological arousal level increased in both age groups, the amplitude of this response was different. There are several possible explanations for this similarity in the response pattern to both emotions. First, anger may co-occur with disgust, possibly because participants perceive the content as morally (and not physically) disgusting^34–36^. This would mean a co-elicitation of emotions occurs. Second, the relevance of the stimuli might have a different effect in each age group, with older adults being more sensitive to situations of social injustice^14,26,37^. This would be the case, for example, for scenes of atrocities committed in the Second World War, which might simultaneously evoke repulsion and anger. Third, anger is the most difficult discrete emotion to induce through MIPs^22^. Regarding affective valence, older adults showed lower affective valence compared to young adults in disgust stimuli. Other studies have found no age-related differences in this self-reported emotion^10^. For sadness stimuli, we found that subjective arousal was higher in older adults, while the physiological response was more intense in young adults (SCL and HR). As with anger and disgust targets, these findings might be explained by the significance of the life experiences in the two age groups. In the case of older adults, it is typically thought they will have more likely experienced a personal loss^10,13,18^ and are more likely to have experienced situations similar to those portrayed in the film clips, with an example being when the content is particularly relevant for older adults such as warlike conflict^11,28,38^.

In contrast, the older participants showed lower levels of subjective emotional valence in the fear stimuli, while the young participants recorded higher heart rate levels. This physiological difference has been reported in previous studies using other MIPs^39^. On the other hand, the older adults identified this emotion significantly less on the DES than the younger ones. Some studies, however, have reported the opposite response^40^.

Concerning our exploration of positive emotions, we also found differences between subjective and physiological emotional systems. On the amusement clips, the younger adults presented higher levels on both parameters and showed coordinated changes across experiential and physiological arousal responses. These data are in contrast to the results on the induction of negative emotions. In addition, the young adults showed higher HR level and perceived the target emotion with higher intensity. This coherence between both systems has previously been observed in a population of young adults following emotion induction using clips eliciting amusement^41^. Also, our findings are consistent with previous research using film clips to study differences between young and older adults^14,26^ In line with the model of strength and vulnerability integration and previous studies suggesting increased positivity in older age, these data appear to provide greater support for the idea that the level of age-related affect depends more on the degree of arousal or activation required by each positive emotion than the emotional category itself. In this case, amusement is a high-arousal positive emotion, and previous studies have suggested the perceived intensity in older adults is lower in response to these types of emotions^42^. However, in the case of lower-arousal emotions, such as enjoyment or happiness, older adults show higher levels of well-being than their younger counterparts^43–45^.

Finally, we found that the tenderness response pattern differed from the amusement emotion. On the tenderness targets, the young participants scored higher on both physiological patterns (HR and SCL), yet their levels of experiential arousal were lower than those of their older counterparts. These findings are interesting as they show that positive emotions should not be included under a generic label of happiness, as has been commonly done in the previous literature^28,31^. To the best of our knowledge, no studies have previously focused on physiological tenderness in older adults. The present study reveals differences between young and older adults in the amplitude of the physiological response pattern and that these differences are reversed in the pattern of subjective responses. We take the current evidence as supporting prominent life span theories. These propose that differences may be due to changes in the way each age group experience the different stimuli and perceive them as more or less pleasant. Various factors may intervene in this process. First, the topics of the clips are key in emotional experience. For example, some of the clips used to elicit tenderness reflected situations of personal risk or social injustices^10^. Second is the relevance of the attachment-related emotions in different life stages, and third, the level of arousal of the different emotions. This is logical in the sense of attending to high-arousal stimuli when young and attending to low-arousal stimuli when older.

In sum, our findings reflect a complex and multiple system, not just a simple response to the affective film clips. Across the analysis of the target emotions, there is a predominant coherence between the different measures of the subjective system and those of the physiological system. However, this coherence is not present in the relation between the two systems. Components of the physiological response and of the subjective response appear to form part of the same emotional phenomenon, but are independent processes^46^. Indeed, the associations between the two systems are usually weaker than expected^47,48^. Thus, our findings suggest that the subjective response and the physiological response form part of a dual-process framework^25^.

With regard to the differences between young and older adults, if we had to speculate, we might attribute these differences to biological or psychological processes. The literature suggests that amygdala dysfunction and neural degradation may affect the interpretation of emotional stimuli. In fact, the aging brain model proposed by Cacioppo et al.^49^ suggests that lower amygdala activation triggers a dampened response to negative stimuli, primarily in the arousal parameter. Moreover, older adults’ autonomic levels are reduced in magnitude^12,50^. Thus, one would expect a dampened response to all negative emotions. Nevertheless, the current study reveals differences in the way each of these emotions is experienced, with fear being the only one that meets such expectations.

With regard to psychological processes, the differences between emotional targets may have various explanations. For example, the physiological emotional response has traditionally been measured during exposure to the mood-inducing stimulus, while the subjective has been assessed after exposure. Then, the subjective response involves cognitive interpretation after watching the clip. In other words, top-down processing (patterns of prefrontal and subcortical neural activity) would be triggered, which could enhance or impair the coherence between the two systems and involve the intervention of different emotion regulation processes^25,51^. At the same time, the motivations, priorities and limitations of each life stage seem to lead individuals to experience different emotions and to experience them with differing intensity. For example, the tendency of older adults to seek goals with a powerful emotional significance and respond more positively to low- or medium-arousal stimuli and more positively to high-arousal emotions^7,52^. In the same line, it has been suggested that the nature of emotional experience in older persons is more complex and mixed than in younger adults^53,54^.

In sum, the age-related differences in emotional reactivity appear to be the result of both changes in the organism and motivational changes. Human behavior is biologically rooted and individuals are flexible in response to challenges and expressions marked by our time. Thus, the relations among the different components of an emotion may follow a synchrony pathway or may situationally uncouple^55^.

As with all research, this work needs to be considered in the light of some limitations. The fact that part of our sample was older adults raises questions about physiological patterns. In this sense, the participants declared that they were taking no medications that might affect the study variables. Nonetheless, they might have been suffering from a hidden pathology, such as auricular fibrillation. Moreover, although multiple autonomic measures were included, an overall representation of the complex functioning of the autonomic nervous system was not achieved.

In future research, the number of measures should be increased, and the simultaneous recording of reactivity and physiological and subjective regulation should be implemented. The literature suggests that both the time and the mode of regulation after a negative emotion differ between young and older persons^56,57^.

In addition, as perceived time is strongly correlated with chronological age, changes in goals appear systematically as people move through adulthood^7^. Nonetheless, future studies should include the measure of socioemotional goals so as to enrich the substantiation of emotional differences between age groups.

As regards the stimuli used in this study, the set contains clips from films released at the end of the 1990s and the start of the twenty-first century, which might suggest a possible impact of generational differences on emotional reactivity. For example, the younger adults might have understood the aggressive style of humor in the amusement clips better than the older adults^58^. In this sense, one of the most common limitations of MIPs is that the stimuli do not elicit only a single emotion. In our work, however, the participants reacted appropriately to the targeted stimuli. Finally, another line of research in relation to the specificity of emotions could be to expand the repertoire of positive emotions in MIPs. Emotions such as tenderness, love or compassion may play a significant role in older adults’ response and emotion regulation.

## Conclusions

This study shows that film clips are an effective tool for eliciting positive and negative emotions in young and older adults. The results support the importance of including physiological emotional variables in studies with older adults and shows that both age groups exhibit different subjective and physiological responses, which are consistent with the idea of decreased biological activation across adulthood. It also demonstrates that the dimensional and discrete models of emotion are complementary and both provide valuable information on the complex emotional system. In this sense, it is important to make advances in the study of how physiological changes in emotional experience may respond to developmental functions that depend on age. Finally, our study furnishes new data on attachment-related emotions in aging and how changes in emotional response are associated with the key motivations of each life stage. It is essential to consider this in order to expand our knowledge of the mechanisms of emotion regulation in older adults and to continue working to enhance their quality of life.

## Method

### Participants

An a priori analysis of the sample required was conducted using G*Power (Version 3.1.9.4.). For an effect size of *F* = 0.17 equivalent to *η*^2^ = .15, taken from the work by Fernández-Aguilar et al. (2018), 0.05 probability of error, 2 groups and 5 measures, the necessary sample size was 66 (33 per age group). We decided to form a sample of 40 per group. Finally, the older adult group comprised 39 participants and the younger group comprised 68, due to the greater availability of volunteers of that age group. The difference in the number of participants per group was taken into account in the variance analysis using robust indicators.

Sixty-eight younger college students (*M* = 18.78, *SD* = 1.71, 69.1% women) and 39 older adults (*M* = 68.51, *SD* = 6.66, 66.7% women) participated in this study. Participants were divided into age groups to form a younger group ranging in age from 18 to 27 and an older group ranging in age from 60 to 84 years. The mean number of years spent in education was 12.71 (0.83) for the younger group and 13.33 (3.08) for the older group [*t*(105) =  − 1.58, *p* = .116, *d* = 0.27]. They were receiving no psychotropic or drug treatment and had no previous history of psychological, psychiatric or neurological disorder, according to the criteria of the Diagnostic and Statistical Manual of Mental Disorders, Fifth Edition (DSM-V) American Psychiatric Association^59^. None of the participants was taking any cardiovascular disease or medical treatment that could affect the results of the task or presented no auditory or visual impairments other than requiring corrective lenses. All were of Caucasian ethnicity and native Spanish speakers. There were no between-group differences in the variables of gender, educational level and mood state prior to the experiment. The participants were recruited from a research volunteer pool at the Department of Psychology at the University of Castilla- La Mancha (UCLM) Medical School, from an association at the *Universidad de Mayores* (a university program for older adults) and two socio-cultural centers in the city of Albacete. The younger adults were all students of courses in psychology at UCLM medical school.

### Measures

Before the experimental session, we administered two questionnaires. The Mini Mental State Examination (MMSE)^60^ was administered to assess cognitive impairment. The MMSE is a screening tool used to measure decline in cognitive abilities such as alterations in memory, where scores of 27 or lower are considered cognitive impairment. No participant was excluded from our study for this reason (*M* = 29.18; *SD* = 0.95). Beck Depression Inventory II (BDI-II)^61^ was also administered. The BDI-II is a self-report questionnaire that assesses symptoms of depression such as anhedonia and sadness. Scores between 10 and 15 are considered to be in a dysphoric range and scores of 16 or above represent a depressed range^62^. The mean scores were 9.25 (5.10) for younger participants and 7.72 (4.59) for older participants [*t*(105) = 1.54, *p* = .124, *d* = 0.31].

During the session, the subjective emotional response was assessed using dimensional and discrete measures. The Self-Assessment Manikin (SAM)^63^ is a self-report questionnaire that assesses emotional response, measuring affective valence, arousal and dominance. Considering the dimensional structure of affect^30^, we administered the items measuring valence and arousal. These two dimensions are those most commonly used in the literature and permit comparison with somato-physiological measures. Thus, participants rated, on a 9-point Likert-type scale, how pleasant (9) or unpleasant (1) and how aroused (9) or relaxed (1) they felt while watching the emotional video clips. The questionnaire uses graphic figures which represent the different emotional states and is therefore rapid and simple to administer in both age groups, regardless of participants’ educational level. The Differential Emotions Scale (DES)^64,65^ was used to assess discrete emotions. This emotional scale has 18 items and uses a 7-point scale (1 = ‘‘not at all’’, 7 = ‘‘very intense’’) to rate the extent to which participants feel each state as they are watching a film clip. The emotional categories used in this study are amusement, anger, disgust, fear, tenderness/love and sadness.

The physiological emotional response was assessed using cardiovascular and electrodermal activity variables. They were measured through blood volume pressure (BVP) and skin conductance (SC), respectively. A novel acquisition system based on an unobtrusive wearable monitoring device and related control software was used to record the signals^66,67^. BVP was estimated using a photoplethysmography (PPG) technique, where a photo-emitter and receiver sensor was in charge of detecting the blood volume fluctuation occurring during the cardiac cycle. Alternations of the BVP waveform are highly correlated with heart ventricular depolarization and repolarization, thus being suitable to measure heart rhythm^68^. The PPG sensor was placed on the index fingertip of the non-dominant hand. The SC signal was acquired by means of a direct current exosomatic technique, using a pair of Ag/AgCl disc electrodes with contact diameters of 10 mm. The electrodes were attached to the middle phalanges on the palmar side of the index and middle fingers. A single-supply, rail-to-rail input/output, instrumental operational amplifier was used to implement a voltage-controlled linear current source. This current is limited to 10 µA/cm^2^ to avoid damage to the sweat gland ducts and to prevent nonlinearities in the current–voltage measurement.

Once data was acquired, BVP and SC signals were processed to reduce noise inherent to the system and to retain useful information. BVP was recorded at a sampling rate of 60 Hz and a 22-bit resolution to perform correct signal acquisition without distortion^69^. Some external causes may severely affect the quality of the BVP signal, such as poor contact of the photo-sensor on the skin, surrounding electrical sources, ambient lights, or changes in temperature, among others. To reduce their negative effect, different filters were applied to the BVP signal. Concretely, baseline drift was removed by carrying out a 0.5 Hz cut-off-high-pass, linear phase finite impulse response (FIR) filter. Then, a 30 Hz cut-off low-pass, linear-phase FIR filter was utilized to eliminate high-frequency noise and power-line interferences. Additionally, peaks related to pulse pumping were located on BVP signals using a robust and reliable peak detection algorithm, capable of dealing with movement artifacts and signal morphologies^70^. Finally, inter-beat intervals (IBIs) were derived from the peak-data series.

Similarly, SC raw data was acquired at a sampling frequency of 10 Hz and 12-bit resolution. These specifications were selected with the aim of preventing distortions and leaving the SC waveform unaltered^71^. SC morphology is the result of two independent components: a fast-changing skin conductance response (SCR), overlapped with a slowly-changing skin conductance level component (SCL). The SCL component ranges from 0 to 0.05 Hz, whilst SCR ranges from 0.05 to 1.5 Hz. Therefore, each SC signal was filtered by applying a 1.5 Hz cut-off low-pass FIR filter to decrease noise generated during the acquisition.

Regarding heart rhythm, the IBI data series were first divided by sixty to obtain the heart rate (HR) measured in beats per minute (BPM). Then, the HR metric was partitioned into five- second equally-separated segments. Finally, the mean of the HR was stored and used subsequently in a statistical analysis. A similar procedure was carried out with the SC processed series. The data series were first divided into equal segments lasting five seconds and the mean of each segment was recorded for subsequent analysis. This methodology is similar to that used by other authors^21,23^.

### Mood induction procedure

We selected fifty-four excerpts from high-definition clips previously validated in a sample of young Spanish adults^72^. The selected scenes maintained the same features used in similar studies^31^. We selected film clips to elicit seven target emotions: sadness, disgust, fear, anger, amusement, tenderness and neutral. The duration of the experiment was approximately one hour, being administered using E-prime 2 Professional (Psychology Software Tools, Inc.). Following the recommendations of previous studies^22^, the experiment was conducted in a room with dimmed lighting on a 27″ computer screen situated in a laboratory (8 m^2^).

In the session, participants started by watching a neutral clip to establish the baseline of the physiological parameters. Each participant watched a set of 9 film clips including 6 emotional targets in a counterbalanced order (sadness, disgust, fear, anger, amusement and tenderness)^28^. They were also required to complete a distraction task to prevent excitation transfer from one emotion to another. The distraction task consisted of distinguishing geometric figures on the screen. At the end of the session, another neutral clip was presented to recover to a relaxed state.

### Data analysis

For each participant, there were three sets of variables: a two-factor subjective evaluation (SAM: valence and arousal, and DES: six discrete emotions) and autonomic response patterns (SCL and HR). The subjective responses were evaluated immediately after each showing of the film clips and the physiological responses were measured on a continuous basis as the experiment was conducted. Furthermore, in order to measure the actual strength of the mood induction for each of the target emotions, the same measures were taken for the initial neutral film clip. For the subjective responses, we considered the mean scores on the SAM and the DES for the neutral stimulus and compared them with those obtained for the emotional stimuli. For the physiological responses, we calculated the mean SCL and HR scores for the neutral clip and compared them with the maximum scores obtained from the emotional stimuli. The statistical analyses were conducted using STATA 15.1^73^, R software (v.3.6.3)^74^ and ggplot2 (v.3.3.0)^75^ for data visualisation.

Previously, we analyzed the possible differences between the groups (young adults vs. older adults) in the responses to the neutral stimulus in order to evaluate any differences in the baseline state. Independent *t* tests were conducted on each of the emotional variables under study (SAM, DES, SCL y HR) and the effect size of the differences was calculated using Cohen’s d test.

To study the age-related differences in identifying each emotional target, we tested it by independent *t* tests. The effect size was also calculated Cohen’s *d* test. To study the ability to identify the target emotions, we ran a discriminant analysis. First, we transformed the direct scores on the DES for each target, assigning a value of 1 to the maximum score and 0 for all other scores). In the case of two or more responses with the maximum score, we assigned a value of 1 to them all. We then performed a 6 × 6 response matrix for the DES and then the discriminant analysis (emotion by emotion; total) to study the degree to which the responses to an emotion or a set of emotions serve to discriminate between the two age groups.

To determine whether the older adults’ physiological and subjective emotional patterns differed from those of their younger counterparts, we ran mixed-design two-way repeated measures ANOVAs. As within-subject variables, we used the neutral film clip measure and that of the corresponding target emotion. As the between-subjects variable, we used the age group (young vs. older adults). We conducted an ANOVA for each target emotion, calculating the valence, arousal, discrete emotion, SCL and HR in younger and older adults. We calculated the effect of the emotion induction (within-subjects), considering the neutral stimulus and the emotional stimuli, the age effect (between-subjects), and the effect of the emotion × age interaction.

### Power analysis

G*Power (version 3.1.9.4) was used to calculate the power of the sample. With the calculation of the *F* test and ANOVA for repeated measures, within-between interaction, taking an effect size value of 0.50 obtained from the work by Fernandez-Aguilar et al.^28^, an *α* value of 0.05, with *n* = 107, divided in two groups, and thirty measure variables (5 measures × 6 target emotions), we obtained a power of 1.00 (1 −* β* err prob), critical *F* = 1.47, numerator *df* = 29.0, denominator *df* = 3,045, and noncentrality parameter λ = 577.8.

### Ethical statement

All participants signed the informed consent prior to the study, according to the requirements of the approved ethics procedure of the Clinical Research Ethics Committee of the Albacete University Hospital and with the Declaration of Helsinki.

## Data Availability

The datasets analyzed within the current study are available from the corresponding author on reasonable request.
